# Methodological and Policy Limitations of Quantifying the Saving of Lives: A Case Study of the Global Fund's Approach

**DOI:** 10.1371/journal.pmed.1001522

**Published:** 2013-10-01

**Authors:** David McCoy, Nele Jensen, Katharina Kranzer, Rashida A. Ferrand, Eline L. Korenromp

**Affiliations:** 1 Centre for Primary Care and Public Health, Queen Mary University London, United Kingdom; 2 Centre for Philosophy, Justice and Health, University College London, United Kingdom; 3 London School of Hygiene & Tropical Medicine, London, United Kingdom; 4 Department of Public Health, Erasmus MC, University Medical Center, Rotterdam, The Netherlands; PLoS Medicine, Canada

## Abstract

David McCoy and colleagues critique the dominance of “lives saved” models of assessing the impact of health programs, using The Global Fund as a case study.

*Please see later in the article for the Editors' Summary*

**Summary Points**

- A recent trend in global health has been a growing emphasis on assessing the effectiveness and impact of specific health interventions.
- For example, it has been estimated that 8.7 million lives were saved between 2002 and mid-2012 by “Global Fund–supported programmes” (as distinct from The Global Fund alone) through antiretroviral therapy (ART); directly observed tuberculosis treatment, short course (DOTS); and distribution of insecticide-treated mosquito nets (ITNs).
- This paper assesses the methods used by The Global Fund to quantify “lives saved,” highlights the uncertainty associated with the figures calculated, and suggests that the methods are likely to overestimate the number of “lives saved.”
- The paper also discusses how the attribution of “lives saved” to specific programmes or actors might negatively affect the overall governance and management of health systems, and how a narrow focus on just ART, DOTS, and ITNs could neglect other interventions and reinforce vertical programmes.
- Furthermore, the attribution of “lives saved” to Global Fund–supported programmes is potentially misleading, because such programmes include an unstated degree of financial support from recipient governments and other donors.

## Introduction

A recent trend in global health has been a growing emphasis on measuring the value of development assistance for health (DAH) and the use of models to estimate the health impact of specific health interventions. Many international donor agencies and global health partnerships now publish quantifiable results that are said to be attributable to their funding and initiatives (see Box 1).

**Box 1. Examples of the Quantification and Attribution of Results and Health Impact to DAH and Specific Health Interventions**

The GAVI (Global Alliance for Vaccines and Immunisation) Alliance claims to have prevented more than 5.5 million child deaths since 2000, through immunizations against vaccine-preventable diseases [1].

The International Development Association reports having immunized 343 million children and given ART to 1.5 million people with HIV [2].

The US President’s Emergency Plan for AIDS Relief (PEPFAR) asserts that “The US directly supported life-saving antiretroviral treatment for nearly 5.1 million men, women and children worldwide” as of September 2012 [3].

According to the STOP TB Partnership, 20 million lives have been saved through TB care and control between 1995 and 2011 [4].

According to the Roll Back Malaria partnership, ITNs and intermittent preventive treatment for pregnant women is estimated to have prevented 842,800 (uncertainty range: 562,800–1,364,645) child deaths due to malaria across 43 malaria-endemic countries in Africa between 2001 to 2010 [5].

According to WHO, UNICEF, and UNAIDS, the introduction of ART has averted 2.5 million deaths in low- and middle-income countries between 1995 and end-2010 [6].

While this may seem sensible at face value, critical examination from at least two angles is needed. First, from a methodological perspective, there are questions about the validity and accuracy of published measures of health impact. Second, from a policy perspective, there are concerns about how the attribution of health impact to *individual* programmes or actors might affect the *overall* governance and management of health systems.

In this Policy Forum article, we critically assess several methodological and policy issues related to the estimation of the number of “lives saved” by The Global Fund to Fight AIDS, Tuberculosis and Malaria (The Global Fund hereafter) as a case study. The Global Fund was established in 2002 to help finance the scale-up of HIV, tuberculosis (TB), and malaria programmes and was chosen as a case study because it is a major provider of DAH and was established explicitly to develop a performance-based funding model.

This paper begins by summarising the published figures of The Global Fund's estimation of “lives saved.” We then describe The Global Fund's approach and methodology for estimating “lives saved.” This is followed by a discussion of three issues: (1) the degree of uncertainty associated with the estimated figures and the potential for bias; (2) the decision to attribute results to “Global Fund–supported programmes” (as distinct from The Global Fund alone) and the double counting of “lives saved” by other agencies; and (3) the implications of measuring the health impact of only a selected number of interventions. This is followed by a discussion that includes certain recommendations.

## The Global Fund's Estimate of “Lives Saved”

According to The Global Fund's 2012 Results Report, 8.7 million lives were saved between 2002 and the middle of 2012 through support for the provision of antiretroviral therapy (ART) to 3.6 million people; directly observed tuberculosis treatment, short course (DOTS) for 17.8 million new cases of TB; and the distribution of 270 million insecticide-treated mosquito nets (ITNs) [7].

The report does not provide a breakdown of the number of “lives saved” by each intervention, nor for each year since 2002. An earlier estimation using a similar methodology indicated that at the end of 2007, DOTS was responsible for 67% of all lives saved by Global Fund–supported programmes, while ART and ITNs contributed 28% and 5%, respectively [8]. However, these proportions will have changed due to the extensive scale-up of ITNs since 2008 leading to greater reductions in mortality due to malaria [9].

## Global Fund's Modelling Approach and Methodology

The Global Fund's estimate of “lives saved” is based on service delivery results consisting of: number of patients alive on ART; number of TB cases placed on DOTS treatment; and number of ITNs distributed to households. The reliability of these results is assumed, with The Global Fund claiming to have established various practices to ensure good reporting by grantees [10] and to have excluded results from countries “with serious data-quality issues.” However, because of The Global Fund's performance-based funding model, grant recipients clearly have strong incentives to over-report performance.

The number of “lives saved” is calculated by comparing the estimated mortality among beneficiaries of ART, DOTS, and ITNs with anticipated mortality rates in a counterfactual “no-treatment/intervention” scenario. The difference in the number of deaths between these two scenarios is taken as an estimate of the number of lives saved by these interventions. Numbers are calculated separately for each intervention and year, and then aggregated into a single figure for publication.

Service delivery results are transformed into estimates of “lives saved” by a modelling exercise that uses additional data on: (1) the quality and effectiveness of ART, DOTS, and ITNs; (2) baseline mortality rates and their causes; (3) the clinical and demographic profile of patients; and (4) treatment compliance and ITN usage rates. Details of the model's methodology for each intervention are summarised in Box 2.

**Box 2. Details of the “Lives Saved” Model for Each Intervention**

**ART**

An epidemiological modelling package (Spectrum) employs demographic and HIV prevalence data and numbers of people on ART to predict trends in HIV-related mortality, incidence and prevalence for each country, with and without ART [6],[11],[12],[13]. Spectrum assumes that ART is only provided to people in need of such treatment, and takes into account the effects of ART on reducing HIV transmission.

Survival rates on ART are derived from cohort studies of people on treatment contained within established databases, including the International Epidemiologic Database to Evaluate AIDS (http://www.iedea.org/). Where previously, Spectrum assumed a standard survival rate of 86% at 12 months after initiation of treatment and 90% for each subsequent year, it now uses region-specific survival rates for adults, which take into account regional estimates of patient profiles in terms of CD4 count, age, and sex at initiation of treatment, and duration of treatment [14]. Under the no-treatment scenario, Spectrum assumes a survival rate of 50% at 12 months and 0% at 24 months, with some region-specific adjustments made for age and sex. For children, who make up around 8% of people living with HIV globally [6], Spectrum uses a standard on-treatment survival rate of 85% for the first year and 93% for each subsequent year [13].

**DOTS**

The model is based on the assumption that every DOTS treatment saves 0.33 lives compared to a no-treatment scenario, as also assumed by WHO’s Stop TB program [4]. This assumed effectiveness is applied to all TB patients and was derived from analyses of various studies and treatment outcome data reported by national TB programmes from different settings [15],[16],[17],[18],[19], thus incorporating variations in HIV and sputum smear status, the proportion of patients with extrapulmonary disease, and treatment completion rates, all of which influence mortality rates.

Up until 2012, most TB programmes only reported the number of newly treated *smear-positive* TB patients to the Global Fund. In order to incorporate treatments of smear-negative and extrapulmonary TB into the model, it is assumed that every smear-positive TB case is accompanied by 0.92 of a case of non-smear–positive TB, a figure derived from smear-positivity ratios that Global Fund–supported programmes routinely report to the WHO [20].

The model does not include any estimation of the dynamic effects of DOTS in reducing TB transmission and thereby reducing mortality through prevention.

**ITNs**

The “lives saved” by ITNs are estimated for countries with stable endemic *falciparum* malaria (i.e., countries in sub-Saharan Africa and Papua New Guinea) [21]. The assumed mortality impact of ITNs is only applied to children aged under 5 years of age, because of a lack of research-based estimates of the impact on mortality in older age groups. Similarly, any “lives saved” by ITNs in other countries are not included because of a lack of evidence of their mortality effect in non-stable endemic areas.

The model assumes that ITNs distributed up to 2008 had an effective lifespan of 1.5 years, but from 2009 onwards, when most ITNs were long-lasting, a lifespan of 3 years was assumed. Each ITN distributed to a household is assumed to result in 0.73 children under 5 years of age sleeping under that ITN during its effective lifespan [22]. The assumed impact on mortality is based on a meta-analysis of five community randomized controlled trials of ITNs conducted in stable malaria settings within four African countries, which estimated an all-cause mortality reduction among children under the age of 5 years of 17% compared to control areas where ITNs were not used at all [23]. The number of “lives saved” is derived by applying the 17% mortality reduction to the estimated child population covered by ITNs and to each country’s average all-cause under-five mortality rate as estimated for 2009.

## Issue 1: Uncertainty and Bias

The methods described in Box 2 depend on a number of extrapolations, assumptions, and generalisations. The number of “lives saved” produced by the model is thus inevitably uncertain.

In the case of ART, while survival rates are adjusted to account for regional variations in CD4 count, age, sex, and duration of treatment [11],[12], the model takes no account of intra-regional variations and assumes a universal standard of treatment quality and adherence, as well as a uniform set of social and economic determinants of health [24],[25], which is far from reality. Reliable measures of the number of patients lost to follow-up after treatment initiation are especially important; but in 2008, only 17 out of 47 countries in sub-Saharan Africa reported data on treatment retention at 24 months [26].

In the case of DOTS, the degree of uncertainty associated with a single assumed mortality effect is illustrated by a meta-analysis of TB case fatality ratios (CFRs) amongst DOTS-treated HIV-negative patients in 19 research studies, which resulted in a pooled CFR of 3.5% but with a 95% confidence interval ranging from 2.5% to 7.2% [27]. Likewise, the assumed 17% child mortality reduction impact of ITNs is held to be true for countries and settings with different baseline mortality rates and patterns of disease. A further reason for uncertainty is the assumed under-five child mortality rates used in the model; in many countries, vital registration data are incomplete and unreliable, and mortality rates have to be derived from population-based surveys that are often conducted once every 5 years [28].

While uncertainty is to be expected from a modelling exercise, the degree of uncertainty is not reported: although uncertainty ranges have been calculated, neither the statistical methods used nor the results are publicly available. However, a paper on the estimated “lives saved” by Global Fund–supported programmes between 2003 and 2007 did publish 95% uncertainty ranges as follows: 619,000 to 774,000 lives for ART; 1.09 to 2.17 million lives for DOTS; and 27,000 to 232,000 lives for ITNs [8].

A more critical question is whether there is any bias towards either under- or overestimating the number of “lives saved.” In the case of ART, assumptions about treatment effectiveness are almost certainly optimistic, having been largely derived from small-scale research studies, clinical trials, and demographic surveillance sites [24],[29],[30] where the quality of care and treatment retention rates are better than in real-world settings.

One meta-analysis of 33 patient cohorts observed in 22 sub-Saharan African countries between 2000 and 2007 [26] found ART retention rates to be as low as 75% at 12 months and 67% at 24 months—lower than the rates assumed in the Spectrum HIV/AIDS epidemic model for Africa. It should also be noted that the rapid increase in size of many ART programmes in recent years has been accompanied by reductions in quality and treatment retention [31]–[34]. In addition, in the few countries that have measured adult mortality trends at a population level during the roll-out of ART (Thailand, South Africa, Botswana, and Malawi), slower and smaller mortality reductions were found than predicted by the Spectrum model [35]–[39].

The assumed mortality impact of DOTS is also likely to be overestimated, primarily because the assumed mortality rate of untreated pulmonary TB (used in the counterfactual “no-treatment” scenario) is based on a meta-analysis of studies from the pre-chemotherapy era of hospitalised patients in the 1930s to 1960s from western Europe and the US. Such patients were mostly detected through passive case finding, and would have consisted of cases of more advanced and severe TB when compared to current cohorts of TB patients who are diagnosed through more active case finding (using improved microscopy and culture), with a higher proportion of less severe cases. Furthermore, untreated TB mortality rates in poor countries from 2000 onwards might be lower than the untreated TB mortality rates of western Europe and the US from 1930 to 1960 due to improvements in living standards and public health.

A further issue about the estimation of “lives saved” by DOTS concerns the use of a “no-treatment” counterfactual. In contrast to ART and ITNs, the scale-up of which corresponded with the establishment of The Global Fund, treatment for TB—including DOTS—was already well established before 2002. Arguably, a more appropriate estimate of “lives saved” by Global Fund–supported programmes would be derived by using a counterfactual based on the coverage and effectiveness of TB treatment in 2002, or on estimated TB mortality rates from 1995, which is the counterfactual used by the WHO Stop TB programme to evaluate the impact of the global DOTS/Stop TB strategy [16]. Using the latter counterfactual would result in a 3- to 4-fold reduction in “lives saved” from TB from the current estimate, reducing the number by several million.

For ITNs, two possible reasons for overestimation in “lives saved” are: the optimistic assumption about the degree to which the number of ITNs distributed to households is translated into actual usage; and the recent scale-up of artemisinin-based combination therapies (ACT) for malaria, which is likely to decrease the relative contribution of ITNs in reducing mortality [40].

Finally, because the model calculates “lives saved” separately for each of the three interventions and then adds these figures together, the total number of “lives saved” may be higher than the actual number of people concerned. For example, an individual co-infected with AIDS *and* TB who receives ART *and* DOTS will be counted as though two lives had been saved. In practice, however, this double counting may be minimal because The Global Fund mainly supports ART provision in southern and East Africa, while its support for DOTS is concentrated in Asian countries [7].

On the other hand, some aspects of the model may underestimate “lives saved.” One is the nonestimation of the effects of ITNs on mortality in children over the age of 5 years and in adults. There is some evidence of reductions in malaria-attributed deaths in older age groups associated with scaled-up ITN distribution [40],[41],[42], and a recent analysis [43] claimed a greater number of deaths due to malaria in individuals over the age of 5 years than previously thought, although adult malaria mortality estimates continue to be disputed [44]. A second aspect is the nonestimation of the effects of ITNs in countries with nonstable endemic malaria. The number of malaria-related deaths in nonstable endemic countries outside Africa in 2010 was recently estimated to total about 104,000 [43], but given the relatively low level of Global Fund support for ITNs in countries outside Africa, the extent of underestimation is likely to be small. Finally, a recent analysis of household surveys across 22 African countries suggests that the effectiveness of ITNs may be slightly greater than that assumed in the model, despite current ITN coverage rates being less than those in the trials on which The Global Fund based its model assumptions [41].

Table 1 summarises the likely causes for both under- and overestimation, assuming the reliability of the data submitted by grant recipients, and suggests an overall overestimation of the number of “lives saved.”

**Table 1:** Potential causes for over- or underestimation of number of “lives saved” in The Global Fund's model.

Intervention	Cause of Underestimation	Cause of Overestimation	Net Effect
**ART**		Optimistic assumptions about ART effectiveness, patient retention and survivalDouble counting of lives saved by ART and DOTS in TB/HIV-co-infected patients	Overestimation
**DOTS**	Noninclusion of the additional dynamic effects of DOTs on reduced TB transmission	Pessimistic assumptions about TB fatality rates in untreated patientsChoice of a “no-treatment” counterfactual	Likely overestimation, especially given choice of counterfactual
**ITNs**	Nonestimation of the effect of ITNs on reducing mortality in children over the age of five and in adultsExclusion of countries outside sub-Saharan Africa and Papua New Guinea	Optimistic assumptions of how grant-reported ITN distributions translate into actual child usage of ITNsLives saved by ITNs will fall if ACT now also reduces malaria deaths	Uncertain, perhaps neutral

## Issue 2: Attribution and Double Counting

A curious feature of the Global Fund's approach to impact assessment is that it estimates “lives saved” by Global Fund–supported programmes and not by The Global Fund alone [8]. In other words, these are “lives saved” by programmes that also receive financial support from other donors and recipient governments, as well as through out-of-pocket payments.

The procedure for attributing results to Global Fund–supported programmes is not entirely clear. According to The Global Fund, patients alive on ART are only counted if three criteria are met: The Global Fund supports an essential element of a country's ART programme (e.g., drug provision or laboratory testing) on a national scale; *and* its HIV grants are deemed to be performing adequately (rated A or B1); *and* The Global Fund has disbursed at least $50 million to the country's HIV programmes in the past 3 years or its total HIV disbursement constituted at least 33% of total reported domestic public expenditure on HIV [22],[45]. A similar set of criteria is used to allocate ITN and DOTS results to Global Fund–supported programmes, although this is not published anywhere.

The scale of the difference between “lives saved” by Global Fund–supported programmes and that of The Global Fund alone can be gauged by comparing the share of global service delivery results for ART, DOTS, and ITNs attributed to Global Fund–supported programmes against The Global Fund's share in global financing for HIV/AIDS, TB, and malaria, as shown in Table 2.

**Table 2:** Global Fund's share in service delivery results and programme financing, low- and middle-income countries.

Disease	Service Delivery Measure	Service Delivery Results		Global Fund Share in Programme Financing, 2010
		Global	Global Fund–Supported Programmes (% of Global Results)	
**HIV/AIDS**	People alive on ART, end-2010	6.65 million [7]	3.0 million (45%) [7]	10% [46]
**TB**	Patients treated under DOTS, for smear-positive TB only, 2010 alone	2.6 million [7]	1.7 million (67%) [7]	11% [20]
	Patients treated under DOTS, all forms of TB, 2010 alone	5.6 million [9]	3.9 million (67%) [9]	
**Malaria**	ITNs distributed, in 2010 alone	145 million [9]	89 million (62%) [9]	45% [47]

Thus, while about 45% of global ART results have been attributed to Global Fund–supported programmes, the contribution of The Global Fund to total AIDS programme financing in low- and middle-income countries was only about 10% [46]. Similarly, while about 68% of global DOTS results and 62% of ITN results were attributed to Global Fund–supported programmes in 2010, The Global Fund's financial contribution to TB and malaria programmes were 11% and 45%, respectively [20],[47].

The financial contribution of Global Fund grants to total health expenditure (THE) in low- and middle-income countries is also relevant because HIV/AIDS, TB, and malaria programmes are significantly reliant on various aspects of the broader health system that are not funded through disease-specific budgets. In 2009, for example, Global Fund disbursements made up only 0.37% of THE across 104 low- and middle-income countries that The Global Fund funded that year [48].

The allocation of results to Global Fund–supported programmes and the subsequent estimation of “lives saved” from these results may easily be misunderstood to reflect the impact of The Global Fund alone. Furthermore, a percentage of those results attributed to Global Fund–supported programmes is also claimed by other donor-supported programmes. For example, at the end of 2010, PEPFAR reported having supported 3.2 million patients, while The Global Fund reported having supported 3 million patients; but the total number of patients supported by PEPFAR and The Global Fund combined was 4.7 million, meaning that 1.5 million patients were claimed to have been supported by both PEPFAR and The Global Fund [49].

## Issue 3: Selectivity

The headline figure of 8.7 million “lives saved” is based on the estimated impact of only three interventions. It excludes Global Fund support for other interventions, including the prevention of vertical HIV transmission; treatment of MDR-TB; HIV testing and counselling; treatment of acute malaria and non-HIV sexually transmitted infections; promotion of condom use; indoor residual spraying; and male circumcision. This selectivity is justified by The Global Fund on the grounds that: (i) there are relatively robust measures of the effectiveness of ART, DOTS, and ITNs on mortality; (ii) ART, DOTS, and ITNs constitute a large part of The Global Fund's overall spending; and (iii) data collection on ART, DOTS, and ITNs is relatively good [8].

Nevertheless, this selective focus may have adverse consequences. It may lead to an overemphasis on ART, DOTS, and ITNs at the expense of other services and interventions. The fact that The Global Fund's 2012–2016 strategy includes numerical targets for delivery of ART, ITNs, and DOTS, but no such targets for other “essential” HIV, TB, and malaria services [9], may be indicative of this.

In theory, The Global Fund could estimate lives saved due to other interventions that have an evidence base of measured mortality reduction. However, data-related deficiencies would result in even greater degrees of uncertainty, and there would be major methodological challenges involved in having to disentangle the separate and unique effects of different interventions on the same population to avoid the double counting involved in “lives saved” calculations.

Regardless of the number of interventions modelled, there are fundamental questions about measuring and attributing “lives saved” to a selection of discrete interventions. The emotive metric of “lives saved” could undermine investment in interventions that are important but which are not easily translated into a measure of saved lives. These include interventions whose impact may involve a prolonged time lag or those which operate through more complex and multi-staged pathways. Examples include many health systems–strengthening investments and interventions aimed at improving the wider determinants of health. It could also undermine investment in interventions that have a greater (and important) impact on morbidity.

The threat that a narrow focus on selected interventions might displace funding and attention from other important interventions is accentuated by the pressure on global health agencies to prove their value. The global health landscape has become crowded with different organisations, diseases, and interventions increasingly competing for global health funding and attention. What matters increasingly is the achievement of a narrow set of concrete, quantifiable, and immediate results, leaving sustainability, equity, long-term capacity development, and comprehensive systems strengthening to be of secondary importance.

The rhetorical power of measures of “lives saved” not only shapes the setting of health priorities and budgets, but also influences how global health actors interact with partners in recipient countries. Ideally, external funding should have an *indirect* impact on health by catalysing national health systems development and supporting ministries of health and other local agencies to perform more effectively. But if external agencies are judged against the delivery and impact of specific interventions, they may encourage vertical programmes and stand-alone systems (over which they can have greater control), and neglect local institution building and national systems strengthening.

These dangers are accentuated by the reporting of results and health impact without any accompanying analysis or assessment of sustainability, efficiency, or equity. Thus, while the last ten years have seen a dramatic improvement in ART, DOTS, and ITN coverage, it is not apparent whether this has been achieved optimally or efficiently (resulting in better health and lower cost), or if lives were saved in ways that improved equity and reduced levels of donor dependency.

## Discussion

This paper argues that the number of “lives saved” that are attributed to Global Fund–supported programmes is not as certain as has been suggested by The Global Fund, and is likely to be an overestimate. Furthermore, estimating the “lives saved” by Global Fund–supported programmes is confusing and potentially misleading, because such programmes include a considerable but unstated amount of financial support from other sources. Finally, a number of potentially negative policy effects are associated with the selective impact estimation of downstream clinical interventions.

While this paper focuses on The Global Fund, the issues raised here apply to other global health partnerships and international donor agencies that are increasingly under pressure to quantify the health impact of their investments. The methods for estimating and attributing “lives saved,” and the consequences of doing so, should be questioned and subjected to critical debate.

In the case of The Global Fund, for a start, greater clarity and explanation about the assumptions and generalisations of the methods are required; this should include publication of uncertainty ranges and of disaggregated estimates of “lives saved” for each of the three interventions and for each year. The Global Fund should also conduct and publish sensitivity analyses, particularly in relation to treatment effectiveness, and publish estimates of “lives saved” through DOTS based on alternative counterfactual scenarios.

If the health impact of ART, DOTS, and ITNs is to be estimated in the form of “lives saved,” we argue that this should not be done as an exercise focused on individual external agencies, but rather on the collective contributions of governments and development partners within countries. This would confer a number of benefits. First, the monitoring of service delivery outputs and the estimation of their health impact would be linked to an assessment of the performance of national health systems (a more appropriate unit for assessment) and the degree to which development partners are working in harmonisation with each other and in alignment with ministries of health and their national plans and priorities. This would help shift more attention towards the strengthening of integrated national plans and information systems.

Second, holistic assessments of service delivery results and health improvement at the country level would allow for a context-based analysis of performance, including assessments of efficiency and equity. This would be aided by cross-country comparisons that would reveal variations in effectiveness (and efficiency) of ART, DOTS, and ITNs that arise from differences in, amongst other things, access to health care, quality of care and treatment adherence, and population coverage of nonclinical determinants of health such as access to clean water and nutrition. By describing this variation, policy attention can be directed not just at the delivery of selected clinical interventions, but also at the social, economic, and environmental conditions that influence the degree to which those interventions are effective. This stands in marked contrast to a modelling approach that assumes standardised levels of effectiveness across countries or regions.

Third, estimates of “lives saved” at the country level might be more valid and less uncertain because they would be derived from more appropriate and country-specific modelling assumptions, and because it would motivate countries to improve the quality of their data. In addition, it could stimulate other actors within countries, such as parliamentary health committees, universities, and local nongovernmental organizations, to develop the capacity to scrutinise the performance of the health system. While many countries produce annual health reports, health needs assessments, and national health plans, which provide some description of progress in the health sector, they are often incomplete or weak. Subnational analyses are frequently absent or superficial; and the fragmented and piecemeal nature of reporting systems, encouraged by vertical and donor-driven DAH, still undermines the development of coherent planning, budgeting, management, and information systems.

While an estimate of “lives saved” by ART, DOTS, and ITNs at country level would still be limited by its narrow focus on three interventions, it would provide a platform for monitoring and evaluating other aspects of HIV, TB, and malaria programmes and be more easily incorporated into a national system of data collection and evaluation that takes into account a wider package of health systems inputs, processes, and outputs, enabling policy makers and planners to consider the importance of investments that do not have a measurable or immediate mortality impact.

If individual external agencies need to estimate their specific contribution to “lives saved,” this could be done more simply by apportioning a share of a country's estimated number of lives saved on the basis of their proportional financial contribution to THE or total HIV/AIDS, TB, and malaria programme financing. This would provide a more meaningful assessment of the contribution of individual agencies, avoid double-counting in reported estimates of “lives saved” by external agencies, and incentivise external agencies to promote coherent national health planning and reporting.

Many of these recommendations (Box 3) are applicable to external agencies in general. However, since 2012, The Global Fund has been providing more active support for detailed national evaluations of programme performance and impact, and more accurate measures of disease incidence, prevalence, mortality, and morbidity in 20 to 25 “high-impact” countries. This provides it with an opportunity to shift emphasis away from estimating “lives saved” by individual interventions and donor-supported programmes, towards an assessment of health systems performance and impact that incorporates all major actors, programmes, and interventions, and a fuller assessment of the contribution of social, economic, and other upstream determinants of health.

**Box 3. Recommendations**

The Global Fund should publish a clear explanation of the assumptions and generalisations of its methods for calculating “lives saved” as well as uncertainty ranges and disaggregated estimates of “lives saved” for each of the three interventions and for each year.

The Global Fund should also conduct and publish sensitivity analyses, particularly in relation to treatment effectiveness, and publish estimates of “lives saved” through DOTS based on alternative counterfactual scenarios.

Estimates of “lives saved” by ART, DOTS, and ITNs should not be focused on individual external agencies, but rather on the collective contributions of governments and all development partners within countries and using country-specific modelling assumptions.

Holistic assessments of service delivery results and “lives saved” at the country level should be accompanied by a context-based analysis of performance, including assessments of efficiency and equity and by cross-country comparisons that would reveal variations in the cost-effectiveness of ART, DOTS, and ITNs.

Should individual external agencies need to estimate their specific contribution to “lives saved,” this could be done more simply by apportioning a share of a country’s overall estimate of “lives saved” on the basis of their proportional financial contribution to THE or total HIV/AIDS, TB, and malaria programme financing.

## Abbreviations

ART: anti-retroviral therapy
ACT: artemisinin-based combination therapies
CFR: case fatality ratio
DAH: development assistance for health
DOTS: directly observed TB treatment, short course
ITN: insecticide-treated mosquito net
PEPFAR: President's Emergency Plan for AIDS Relief
THE: total health expenditure
